# Comparative Evaluation of Total Ergot Alkaloids Determination by ELISA and UHPLC-MS/MS in Wheat: Implications of Cross-Reactivity and Matrix Effects

**DOI:** 10.1021/acs.jafc.6c05234

**Published:** 2026-06-10

**Authors:** Chamali Kodikara, Dainna Drul, Nandika Bandara, Sheryl A. Tittlemier

**Affiliations:** † Grain Research Laboratory, 56032 Canadian Grain Commission, 1404-303 Main Street, Winnipeg, Manitoba R3C 3G8, Canada; ‡ Department of Food & Human Nutritional Sciences, 8664 University of Manitoba, Winnipeg, Manitoba R3T 2N2, Canada; § Richardson Centre for Food Technology and Research, University of Manitoba, Winnipeg, Manitoba R3T 2N2, Canada

**Keywords:** ergot alkaloids, ELISA, UHPLC-MS/MS, cross-reactivity, matrix effects, cereal grains, screening methods

## Abstract

Ergot alkaloids (EAs) are toxic secondary metabolites found in cereal grains that require effective monitoring strategies. This study evaluated a commercially available enzyme-linked immunosorbent assay (ELISA) for total ergot alkaloids against a validated ultrahigh-performance liquid chromatography-tandem mass spectrometry (UHPLC-MS/MS) reference method using naturally contaminated wheat and durum samples. Beyond method comparison, the study examined cross-reactivity and matrix effects to explain differences between immunoassay and chromatographic measurements. The ELISA demonstrated good repeatability and calibration stability across kits and days, but systematic bias relative to UHPLC-MS/MS was observed, with ELISA results generally underestimating chromatographic totals. ELISA accuracy assessment using certified reference and proficiency test materials revealed inconsistent bias, including occasional overestimation. ELISA cross-reactivity, based on half-maximal inhibitory concentration (IC_50_), revealed compound- and matrix-dependent immunochemical effects, explaining the observed discrepancies. These findings support ELISA as a high-throughput screening and prioritization tool while confirming that definitive quantification requires UHPLC-MS/MS confirmation for regulatory purposes.

## Graphical abstract

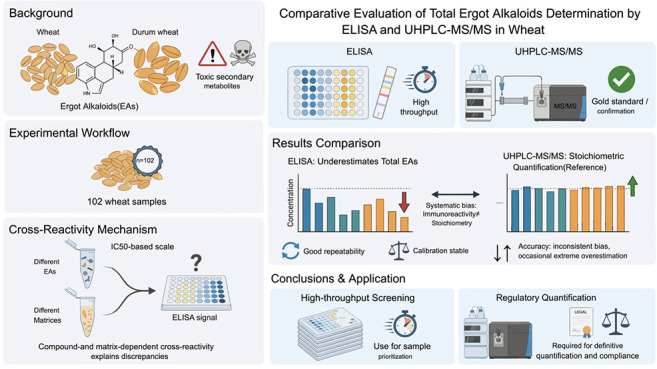

## 1 Introduction

Ergot alkaloids (EAs) are a structurally diverse group of indole-derived mycotoxins produced primarily by phytopathogenic fungi of the genus *Claviceps*, most notably *Claviceps purpurea*. These fungi infect the flowering heads of cereal crops, replacing the developing grain with hardened fungal structures (sclerotia) that contain ergot alkaloids. Cereals such as rye, wheat, barley, oats, and triticale are particularly susceptible, with infection severity strongly influenced by environmental conditions during flowering, including rainfall and humidity.^1–3^ Although improvements in agronomic practices and grain cleaning have reduced the prevalence of visible ergot sclerotia, ergot alkaloids can persist in cereals and cereal-based foods even in the absence of detectable sclerotia. This may occur because sclerotia can fragment during harvesting, handling, cleaning, or milling, producing small particles that are not visually detected but may still contribute ergot alkaloids to grain or processed products, continuing to pose food and feed safety concerns.^4,5^ Ergot alkaloids share a common tetracyclic ergoline backbone and occur as structurally related compounds, including ergometrine, ergotamine, ergosine, ergocristine, ergocryptine, and ergocornine, together with their corresponding C-8 epimers. These epimers occur as *R-* and *S-*configurations, commonly referred to as -ine and -inine forms, respectively. Epimerization at the C-8 position can occur during fungal metabolism and during sample extraction, storage, and analysis, complicating accurate quantification.^6,7^ Although S-epimers were historically considered biologically inactive, recent evidence indicates that they may contribute to overall exposure and toxicity, supporting their inclusion in analytical and regulatory frameworks.^3^

Dietary exposure to elevated levels of ergot alkaloids has been associated with a range of adverse health effects, collectively referred to as ergotism, including vasoconstriction, ischemia, neurological disturbances, and hallucinations.^3^ These effects arise primarily from interactions with adrenergic, serotonergic, and dopaminergic receptors.^8–10^ In response, regulatory limits for ergot alkaloids in food have been introduced or revised in several jurisdictions. In the European Union, maximum levels have been established for the sum of 12 ergot alkaloids in cereal milling products under Regulation (EU) 2021/1399, which was subsequently consolidated under Regulation (EU) 2023/915. This sum includes ergometrine, ergosine, ergotamine, ergocornine, ergocryptine, and ergocristine together with their corresponding -inine epimers, increasing the demand for reliable analytical methods to support routine monitoring and enforcement.^1,11^

Liquid chromatography-tandem mass spectrometry (LC-MS/MS), particularly ultrahigh performance liquid chromatography-tandem mass spectrometry (UHPLC-MS/MS), is widely regarded as the reference technique for confirmatory determination of ergot alkaloids due to its sensitivity, selectivity, and ability to resolve epimeric forms. Recent methods routinely achieve low microgram-per-kilogram limits of quantification across a range of cereal matrices.^12–17^ However, LC-MS/MS approaches are resource-intensive and typically confined to centralized laboratories, limiting their suitability for high-throughput screening or rapid decision-making in grain-handling and quality-control settings. Consequently, rapid screening methods, most notably enzyme-linked immunosorbent assays (ELISAs), are commonly employed as complementary tools to chromatographic analysis.^18–21^ ELISAs offer reduced analysis time, simplified workflows, and lower per-sample costs, making them attractive for preliminary screening and sample triage.^22–24^

In contrast to other regulated mycotoxins, however, the commercial availability of ELISAs for ergot alkaloids is limited. While multiple kits have been evaluated, performance has varied substantially across assays and matrices.^1^ At present, the Randox Food Diagnostics Ergot Alkaloids ELISA is the only widely marketed microplate ELISA that quantifies the sum of 12 major ergot alkaloids, comprising ergometrine, ergosine, ergotamine, ergocornine, ergocryptine, and ergocristine together with their corresponding -inine epimers, consistent with the analyte scope used for EU maximum levels for ergot alkaloids in specified cereal products.^25^

In addition, cereal matrix effects may influence antibody binding and signal generation, potentially resulting in biased quantification relative to LC-MS/MS reference methods. These limitations are particularly relevant given the compositional variability of ergot alkaloids across cereal species, geographic regions, and growing conditions.^12^ Recent reviews emphasize the need for systematic, matrix-specific evaluation of immunoassay performance before such methods can be confidently integrated into regulatory or industrial monitoring programs.^1,6,11^ However, independent evaluations of commercially available ergot alkaloid ELISAs, particularly those benchmarked against validated UHPLC-MS/MS methods using naturally infested wheat samples, remain limited.

In this context, we hypothesized that measurements of total ergot alkaloids obtained using a commercially available ELISA are influenced by matrix effects and antibody cross-reactivity, resulting in systematic differences compared to UHPLC-MS/MS, which provides compound-specific quantification. To test this, the objective of the present study was to conduct a comprehensive performance evaluation of a commercially available ELISA for the detection of ergot alkaloids in wheat, using UHPLC-MS/MS as a reference method. Specific aims included assessment of repeatability and intermediate precision, extraction efficiency and extract stability, the influence of test portion mass, method accuracy relative to UHPLC-MS/MS, and characterization of antibody cross-reactivity toward individual ergot alkaloids in both solvent and wheat matrix. The results provide data-driven guidance on the appropriate use of ELISA-based screening within tiered analytical strategies for regulatory monitoring and food safety decision-making.

## 2 Materials and Methods

### 2.1 Chemicals, Standards, and Reagents

Acetonitrile, methanol, and formic acid (99%) of HPLC grade were^26^ obtained from Fisher Scientific (Thermo Fisher Scientific, Mississauga, ON, Canada). Ammonium carbonate (LC-MS grade) was used to prepare extraction solvents and mobile phases for UHPLC-MS/MS analysis and was obtained from Fisher Scientific. Ultrapure water was produced using a Milli-Q water purification system (Millipore, Billerica, MA, USA). Ergot alkaloid analytical standards, including ergometrine, ergotamine, ergosine, ergocristine, ergocryptine, ergocornine, and their corresponding C-8 epimers (ergometrinine, ergotaminine, ergosinine, ergocorninine, ergocryptinine, and ergocristinine), and the internal standard dihydroergotamine were obtained from Romer Laboratories/Biopure (Tulln, Austria).^26^ Total ergot alkaloids were determined using a commercially available competitive enzyme-linked immunosorbent assay (ELISA) kit (Randox Food Diagnostics Ltd., Crumlin, UK; Ergot Alkaloids ELISA, EA3491). The kit included antibody-coated microtiter plates, enzyme-labeled conjugate, calibration standards, conjugate diluent, wash buffer concentrate, substrate solution, and stop solution. Calibration standards supplied with each kit lot (0, 0.29, 0.67, 1.60, 3.87, and 8.00 μg/kg) were used to generate run-specific calibration curves.

### 2.2 Samples and Reference Materials

Wheat samples used in this study (*n* = 102) were obtained from the CGC cargo monitoring program and consisted of export cargo samples collected during 2018, 2021, and 2022. Export cargo samples were collected through CGC sampling procedures used at licensed grain handling facilities for grain destined for export. Samples were selected from archived materials for which corresponding UHPLC-MS/MS data and sufficient ground material for ELISA analysis were available; therefore, the selected years reflect sample availability rather than a continuous annual series. For this study, archived ground sample material was subsampled to obtain 2.5 g test portions for ELISA analysis according to the manufacturer’s protocol. Corresponding UHPLC-MS/MS results were obtained using the validated CGC reference method described in Section 2.3, in which 10 g test portions were extracted, and total ergot alkaloid concentration was calculated as the sum of 12 quantified alkaloids: ergometrine, ergosine, ergotamine, ergocornine, ergocryptine, and ergocristine, together with their corresponding-inine epimers.^26^ The samples originated from multiple geographic regions across Western Canada and spanned a wide range of ergot alkaloid concentrations, from below detection limits to approximately 800 μg/kg total ergot alkaloids. In addition, six independent ground wheat samples with previously established total ergot alkaloid concentrations by UHPLC-MS/MS were used to evaluate ELISA repeatability, extraction performance, extract stability, and test portion mass effects. For these experiments, replicate test portions were subsampled from each ground sample as laboratory replicates. Certified rye reference materials (*n* = 3; Aokin, Berlin, Germany) with assigned total ergot alkaloid values and wheat proficiency test materials (*n* = 2) were included to support accuracy assessment and quality control. In-house wheat reference materials (IHRM) with characterized concentrations ranging from 107 to 665 μg/kg of total ergot alkaloids were prepared and analyzed in each ELISA run to monitor intermediate repeatability and analytical consistency.

### 2.3 UHPLC-MS/MS Reference Method

UHPLC-MS/MS analysis of ergot alkaloids was performed using the validated Canadian Grain Commission (CGC) method described by Tittlemier et al. (2015). Briefly, ground grain (10 g) was extracted with 50 mL of acetonitrile/aqueous ammonium carbonate (84:16, v/v; 3.03 mM ammonium carbonate) to provide alkaline extraction conditions, then shaken for 30 min and centrifuged at 2450*g* for 10 min at room temperature. An aliquot of extract (464 μL) was combined with internal standard solution (dihydroergotamine; 20 μL of 4 ng/μL) and aqueous ammonium carbonate (1536 μL), vortex-mixed, and filtered through a 0.45 μm polytetrafluoroethylene syringe filter before injection into the UHPLC-MS/MS system. As in the validated reference method, dihydroergotamine was added after extraction to correct for postextraction analytical variability; extraction recovery was evaluated during method validation. Chromatographic separation was achieved using a reversed-phase BEH C18 column (2.1 × 100 mm, 1.7 μm; Waters, Milford, MA, USA) with the autosampler maintained at 15 °C and the column at 30 °C. Mobile phases consisted of (A) 3.03 mM aqueous ammonium carbonate and (B) acetonitrile. The gradient program was as follows: initial, 75% A/25% B; 1.00 min, 60% A/40% B; 5.00 min, 40% A/60% B; 8.00 min, 22% A/78% B; 10.50–11.00 min, 10% A/90% B; and 13.00–16.00 min, 75% A/25% B, with a total run time of 16 min. Detection was performed using positive electrospray ionization in multiple reaction monitoring mode on a Waters Xevo triple quadrupole mass spectrometer (Waters, Milford, MA, USA). Under the validated chromatographic conditions, dihydroergotamine did not coelute with ergotamine, ergotaminine, or the other target ergot alkaloids. Identification and quantification were based on retention-time criteria and compound-specific MRM transitions, thereby minimizing potential isotopic or coelution interference. Precautions to minimize epimerization, including controlled handling of standards and extracts and reduced light exposure, were followed as described in the reference method. UHPLC-MS/MS quantification was performed using analyte-specific, matrix-matched calibration curves prepared according to the validated CGC reference method, with calibration levels of 0.046, 0.2, 1, 2, 10, and 20 μg/kg. Dihydroergotamine was used as the internal standard, and concentrations were calculated from internal-standard-normalized analyte responses with method dilution factors applied. Total ergot alkaloid concentration was calculated as the arithmetic sum of all quantified ergot alkaloids and epimers included in the validated method.^26^

### 2.4 ELISA Procedure

Total ergot alkaloids were determined using the Randox competitive ELISA according to the manufacturer’s protocol unless otherwise stated. The assay is based on competition between free ergot alkaloids in the sample extract and enzyme-labeled ergot alkaloid conjugate for binding to antiergot alkaloid antibodies immobilized on a microtiter plate. Ground wheat samples (2.5 g unless otherwise specified) were extracted with 25 mL of methanol/water (60/40, v/v) containing 0.1% formic acid by vortex-mixing for 1 min, followed by shaking for 15 min. Extracts were clarified by centrifugation at 1500*g* for 2 min. Clarified extracts were diluted with the kit wash buffer before analysis, resulting in a 100-fold dilution for wheat, as specified by the manufacturer. Calibration standards supplied with the kit were included in each run to generate run-specific calibration curves. The ELISA calibration curve was generated using the kit-supplied standards at 0, 0.29, 0.67, 1.60, 3.87, and 8.00 μg/kg, and prepared in the manufacturer-provided assay matrix/buffer; no wheat matrix-matched calibration curve was used for routine sample quantification. Sample extracts, standards, and quality control materials were analyzed in duplicate. Quality control materials included a blank wheat extract as a negative control and in-house wheat reference materials with previously characterized concentrations of ergot alkaloids as positive controls. Following the addition of conjugate and incubation at room temperature (15–25 °C) in the dark, plates were washed to remove unbound reagents. Color development was initiated by adding the substrate solution and terminated by adding the stop solution. Absorbance was measured at 450 nm using a microplate reader (BioTek 800 TS Absorbance Reader, Agilent, CA, USA). Calibration curves were constructed using four-parameter logistic regression (4PL), and sample concentrations were interpolated and corrected for dilution factors.

### 2.5 ELISA Experimental Design

Randox competitive ELISA was evaluated for accuracy and precision and compared against a UHPLC-MS/MS method. Various factors affecting ELISA accuracy and precision were investigated.

#### 2.5.1 Accuracy

The accuracy of the ELISA method for the determination of total ergot alkaloids was evaluated by comparison with a validated UHPLC-MS/MS reference method. Accuracy assessment followed a multitiered approach that incorporated real-world samples, certified reference materials (CRMs), and proficiency test materials to ensure accuracy across different matrices and concentration ranges. A total of 102 wheat and durum cargo monitoring samples, previously analyzed by the CGC UHPLC-MS/MS method, were used as samples for accuracy evaluation. These samples spanned a range of total ergot alkaloid concentrations (approximately 0–800 μg/kg), allowing assessment of ELISA performance at various levels. Duplicate aliquots from the extract of one test portion were analyzed by ELISA, and mean ELISA values were compared directly with corresponding UHPLC-MS/MS results to evaluate ELISA accuracy and agreement with the reference method under the applied analytical workflow. Duplicate independent test portions were not used because the objective of this comparison was method agreement rather than estimation of sampling or subsampling error. To further evaluate ELISA accuracy, three rye CRMs with known ergot alkaloid concentrations and two external proficiency test materials (wheat) were included in the study. These materials were analyzed using the same ELISA procedure as for routine samples, and the measured concentrations were compared with their certified or assigned values. This approach allowed evaluation of ELISA accuracy and agreement with the UHPLC-MS/MS reference method under the applied analytical workflow. This approach allowed evaluation of ELISA accuracy and agreement with the UHPLC-MS/MS reference method under the applied analytical workflow.

#### 2.5.2 Precision

Interday repeatability between production lots was performed using six wheat samples previously characterized by UHPLC-MS/MS (63, 125, 337, 347, and 409 μg/kg total ergot alkaloids), which were analyzed in replicate (*n* = 4) test portions using two different ELISA production lots. Mean total ergot alkaloid concentrations were compared between lots.

#### 2.5.3 Extraction Thoroughness (Single vs Sequential Extraction)

Laboratory replicate test portions independently subsampled from each ground wheat sample (*n* = 4 per sample; five samples from 2.6.2) were independently extracted using 25 mL of methanol/water (60/40, v/v) containing 0.1% formic acid, according to the kit instructions. A second treatment used two sequential extractions: after centrifugation, the supernatant was removed as completely as possible, a fresh 25 mL aliquot of extraction solvent was added, extraction was repeated, and the two extracts were combined. The increased final extract volume from the sequential extraction was accounted for when calculating sample concentrations, and the results were expressed on the original sample-mass basis. For statistical comparison of extraction approaches, replicate test portions were first averaged within each sample and extraction condition, and the paired sample means were compared between single and sequential extraction.

#### 2.5.4 Extract Stability During Refrigerated Storage

Extracts from the repeatability experiment were analyzed on the day of extraction and then stored for 24 h at 5 °C, after which ELISA analysis was repeated. Mean concentrations (*n* = 4 per sample) were compared between time points.

#### 2.5.5 Effect of Test Portion Mass

Two wheat samples previously characterized by UHPLC-MS/MS (∼255 μg/kg and ∼715 μg/kg) were analyzed using test portions of 2.5, 5.0, 10, and 20 g (*n* = 5 per test portion), following the kit instructions. The 2.5 g test portion represents the manufacturer-recommended routine sample mass; therefore, the experiment was designed to evaluate whether increasing test portion mass above the kit protocol affected ELISA response or variability. Means and variances were compared across portion masses. To support ongoing quality monitoring and assess intermediate repeatability across a larger number of analytical days, an IHRM (75 μg/kg) was analyzed in each run using the routine ELISA test portion mass of 2.5 g.

### 2.6 Cross-Reactivity Characterization

Cross-reactivity toward individual ergot alkaloids was assessed in both solvent and wheat matrix. For solvent-based cross-reactivity, individual solutions of 12 ergot alkaloids were prepared in extraction solution (60:40:0.4, v/v/v, methanol:water:formic acid) diluted into wash buffer (100/900, v/v) and analyzed using the Randox ELISA on the same day to reduce procedural variance; each alkaloid was tested in duplicate across 0, 0.29, 0.67, 1.60, 3.87, and 8.00 μg/kg. For matrix-based cross-reactivity, a blank wheat extract verified to be free of ergot alkaloids by ELISA and UHPLC-MS/MS was spiked with the same 12 alkaloids over the same concentration range (0, 0.29, 0.67, 1.60, 3.87, and 8.00 μg/kg) and analyzed. Cross-reactivity was quantified using an IC_50_-based approach consistent with the method described by Tangni et al. (2010) for immunoassay specificity evaluation:^27^ dose–response data were used to obtain the IC_50_ (the concentration producing a 50% signal reduction relative to the zero standard), and relative cross-reactivity was calculated as the ratio of IC_50_ values between the reference analyte and the tested analyte × 100.^27^ In this study, the IC_50_ ratio approach was applied using ergotamine as the reference alkaloid because ergotamine is one of the major regulated ergot alkaloids, produced a reproducible sigmoidal response within the assay working range, and provided a suitable reference point for expressing relative antibody cross-reactivity among structurally related ergot alkaloids and epimers.

### 2.7 Statistical Analysis

ELISA calibration curves were generated using four-parameter logistic (4PL) regression, as recommended for competitive immunoassays, using absorbance values obtained from the kit calibration standards (0, 0.29, 0.67, 1.60, 3.87, and 8.00 μg/kg). Sample concentrations were interpolated from the fitted calibration curve, corrected for the appropriate dilution factor, and expressed as total ergot alkaloid concentrations in μg/kg.

Method precision was assessed using replicate measurements of the test portions from each sample and is reported as mean ± standard deviation. Interday and intermediate repeatability were evaluated using relative standard deviation (RSD, %) across different ELISA kit lots and analytical days, respectively.

Agreement between ELISA and UHPLC-MS/MS was evaluated using linear regression and Bland-Altman analysis, consistent with established approaches for comparing screening and reference analytical methods. Linear regression was performed by plotting mean ELISA results against corresponding UHPLC-MS/MS results for wheat and durum samples (*n* = 102). The coefficient of determination (R^2^) was used to assess the strength of association, while the slope and intercept were examined to identify proportional and constant bias, respectively. Because UHPLC-MS/MS was treated as the reference method, deviations of the regression slope from unity were interpreted as evidence of proportional bias in ELISA response. Bland-Altman analysis was used to further assess agreement between methods by plotting the difference between ELISA and UHPLC-MS/MS results against the mean of the two methods for each sample. The mean difference (bias) and limits of agreement, defined as the mean difference ± 1.96 times the standard deviation of the differences, were calculated to evaluate systematic bias and concentration-dependent trends.

To evaluate ELISA performance factors, ELISA-measured total ergot alkaloid concentrations were compared among experimental groups, including kit production lot, extraction approach, extract storage time, and test portion mass. For two-level comparisons, including kit lot, single versus sequential extraction, and Day 1 versus Day 2 extract analysis, two-sample *t* tests were used. For Day 1 versus Day 2 extract stability comparisons, paired *t* tests were used because the same extracts were analyzed before and after storage. For experiments involving more than two groups, including test portion mass, one-way ANOVA was used to compare mean ELISA concentrations among treatment levels within each sample. Homogeneity of variance among treatment groups was assessed using Levene’s test based on absolute deviations. Differences were considered statistically significant at *p* < 0.05. All statistical analyses, including calibration modeling, regression analysis, Bland-Altman evaluation, and descriptive statistics, were performed using OriginPro 2025b (OriginLab Corporation, Northampton, MA, USA).

## 3 Results and Discussion

### 3.1 Evaluation of ELISA Precision and Procedural Factors

The performance of the commercial ELISA was first evaluated for analytical precision and procedural robustness, focusing on lot-to-lot variability, extraction efficiency, extract stability, and test portion mass (Figures 1–4). Across five naturally infested wheat samples spanning a broad concentration range of approximately 46–360 μg/kg total ergot alkaloids as measured by ELISA, no statistically significant differences were observed between two independent ELISA lot packages (Figure 1). Between-lot differences were not statistically significant (*p* > 0.05), indicating that the two ELISA kit lots evaluated produced comparable responses under the tested laboratory conditions. This finding supports short-term lot-to-lot consistency for the lots examined, although broader interlot performance would require evaluation of additional production lots. This finding is notable given that antibody-based assays are inherently sensitive to manufacturing variability, particularly differences in antibody affinity distributions and conjugate activity. Comparable interlot precision has been reported for well-controlled mycotoxin ELISAs when production consistency is maintained, supporting their use in routine screening programs.^28^

**Figure 1:**
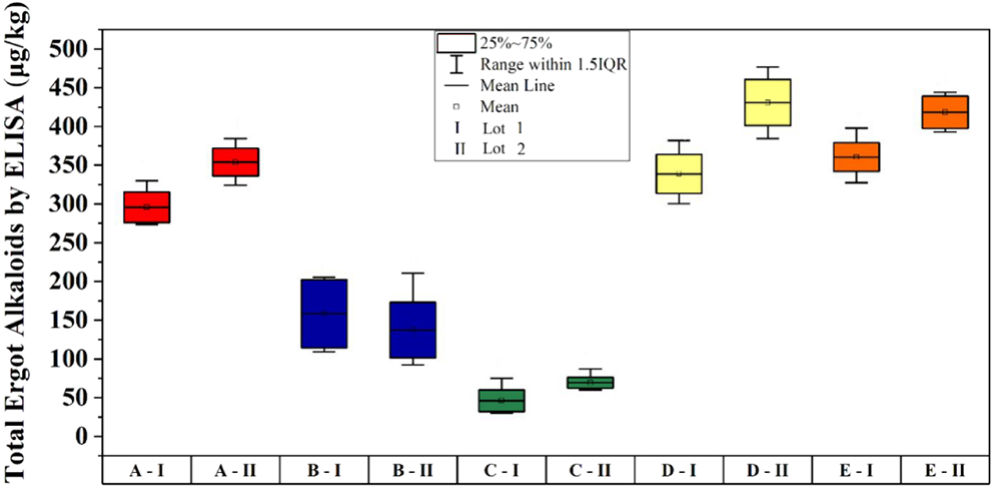
Interday and lot-to-lot repeatability of the ELISA method for analysis of five samples (A-E). Comparison of total ergot alkaloid concentrations determined by the commercial ELISA using two different production lots of the kit (Lot I and Lot II). Each box represents the distribution of four test portion replicate measurements; the solid line inside each box indicates the median, the box limits represent the 25th–75th percentiles (interquartile range), and whiskers extend to 1.5 × the interquartile range.

**Figure 2:**
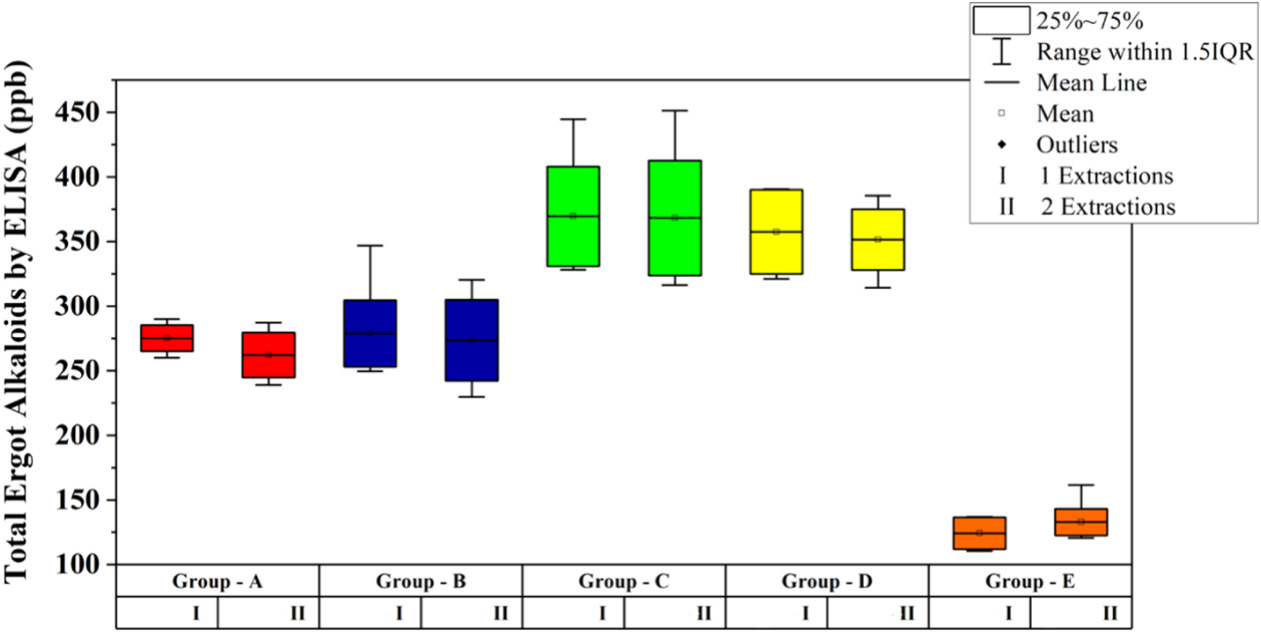
Comparison of total ergot alkaloid concentrations obtained by ELISA following a single extraction and a sequential extraction (two successive extractions combined) of five wheat samples (A–E). Each box shows the distribution of four laboratory replicate test portions independently extracted for each sample and extraction condition. Statistical comparison of extraction approaches was performed using paired sample-level means; the solid line inside each box indicates the median, the box limits represent the 25th–75th percentiles (interquartile range), and whiskers extend to 1.5× the interquartile range. Statistical comparison of extraction approaches was performed using paired sample-level means; no statistically significant difference was observed between single and sequential extraction (*p* > 0.05).

**Figure 3:**
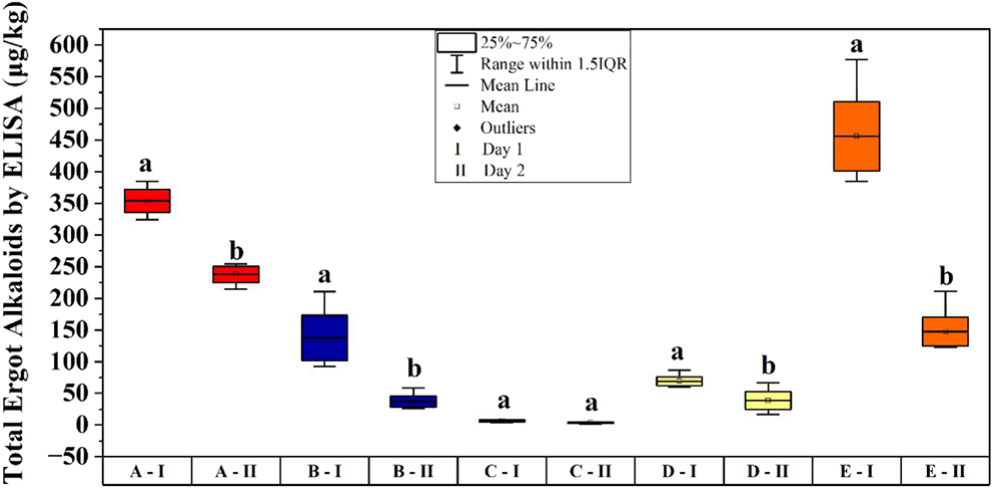
Total ergot alkaloid concentrations measured by ELISA for extracts from five wheat samples analyzed immediately after extraction (Day 1) and after storage at 5 °C for 24 h (Day 2). Each box represents the distribution of four test portion replicate measurements; the solid line inside each box indicates the median, the box limits represent the 25th–75th percentiles (interquartile range), and whiskers extend to 1.5× the interquartile range. Statistical comparison between Day 1 and Day 2 within each sample was performed using paired *t* tests; significant decreases were observed after 24 h storage at 5 °C (*p* < 0.05).

**Figure 4:**
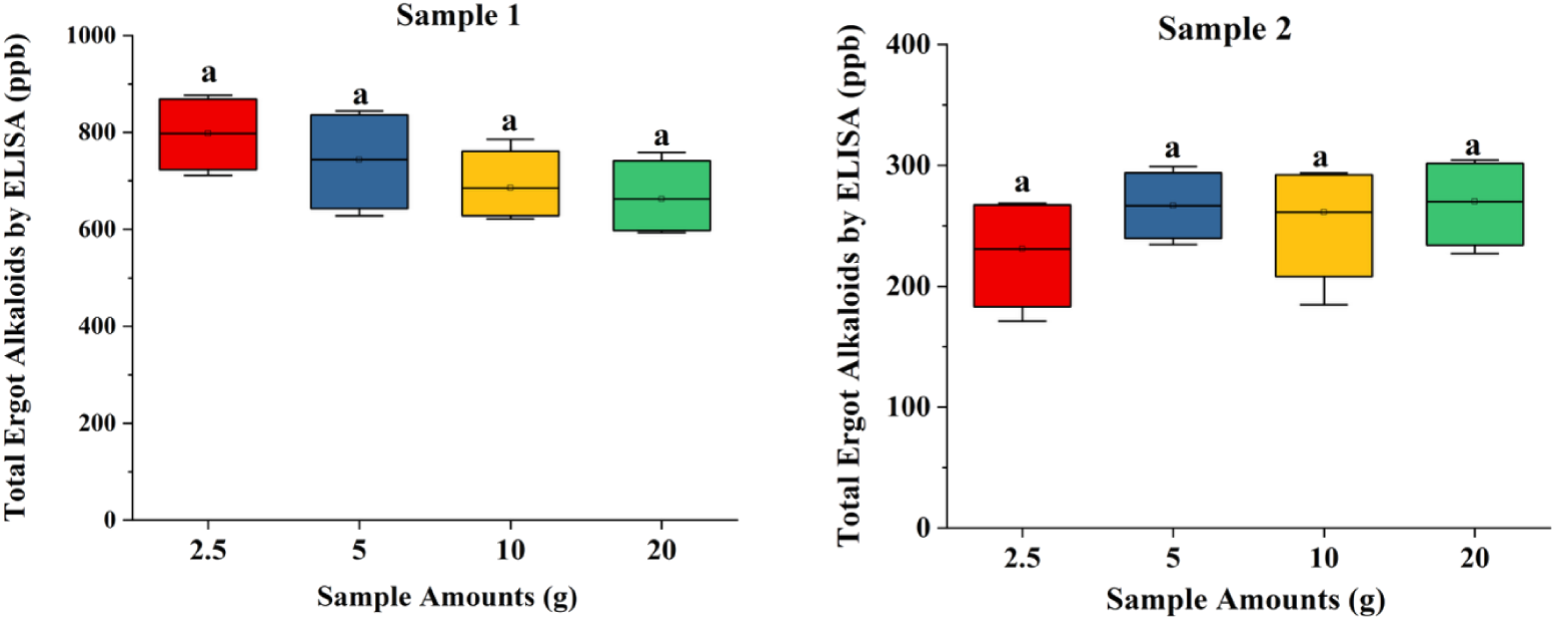
Effect of test portion mass on total ergot alkaloid concentrations measured by ELISA for two wheat samples. Each box represents the distribution of four test portion replicate measurements; the solid line inside each box indicates the median, the box limits represent the 25th–75th percentiles (interquartile range), and whiskers extend to 1.5× the interquartile range. The box plots in each graph differ by letter when the mean recovery percentages differ significantly (*p* < 0.05) according to Tukey’s test.

Extraction thoroughness within the ELISA was assessed by comparing the manufacturer-recommended single extraction with a sequential extraction approach combining two extracts (Figure 2). Across five wheat samples, mean ELISA concentrations obtained following sequential extraction were not statistically different from those obtained using a single extraction when compared using paired sample-level means (*p* > 0.05). Differences between extraction approaches were generally within ±10%, indicating that the single extraction protocol was sufficient to recover the fraction of ergot alkaloids that is immunoreactive under the kit’s specified extraction and dilution conditions. Sequential extraction was not evaluated using UHPLC-MS/MS in the present study because the UHPLC-MS/MS extraction procedure was applied as part of the previously validated CGC reference method. Similar behavior has been reported for other commercial mycotoxin ELISAs, where additional extraction steps did not significantly change measured concentrations when solvent composition and dilution ratios were maintained within the assay’s validated operating window, supporting the adequacy of single-step extraction for screening applications.^28,29^ For ergot alkaloids specifically, previous work has shown that systematic differences between ELISA and UHPLC-MS/MS totals arise primarily because of the different measurement principles of ELISA (immunoreactivity-based detection) and UHPLC-MS/MS (compound-specific quantification).^30^

In contrast to the extraction and test portion mass, extract stability had a significant effect on ELISA response (Figure 3). When wheat extracts were stored at 5 °C for 24 h and reanalyzed by ELISA, measured total ergot alkaloid concentrations decreased significantly for five samples (*p* < 0.05), with reductions of approximately 15–35% relative to the first day-of-extraction measurements. This experiment was designed to evaluate the operational stability of ELISA-ready extracts under the kit workflow, rather than to determine the chemical stability of individual ergot alkaloids. Therefore, UHPLC-MS/MS analysis was not performed on the stored ELISA extracts. The observed decrease can be interpreted as a reduction in ELISA-measured immunoreactivity after storage, which could reflect changes in analyte availability, antibody recognition, or extract/matrix interactions, rather than confirmed degradation of individual alkaloids.

Varying the test portion mass from 2.5 to 20 g did not result in changes in mean ELISA concentrations or variance (Figure 4). Two samples with total EAs (≈255 and 715 μg/kg) were analyzed across 4 different test portion masses, and the mean concentrations were not different at each level (*p* > 0.05). This suggests that, within this range, the extraction solvent volume and subsequent dilution steps specified by the kit are sufficient to buffer differences in sample mass, minimizing subsampling effects. In addition, homogeneity of variance was confirmed across portion masses: Levene’s tests (absolute deviations) indicated no statistically significant differences in variance among mass levels (F­(3,12) = 0.88, *p* = 0.48; F­(3,12) = 0.61, *p* = 0.61). Together, these results indicate that within the evaluated range, changing test portion mass did not measurably alter either the central tendency (mean response) or the dispersion (variability). The variability observed across mass levels likely reflects the combined contributions of subsampling heterogeneity, differences in extraction efficiency, and routine ELISA assay and measurement variability, rather than any systematic effect of test portion size. Similar observations have been reported for ELISA-based screening of other mycotoxins, where test portion mass has limited influence, provided that extraction and dilution ratios remain within the assay’s optimized design window.^29^

Taken together, the results presented in Figures 1–4 demonstrate that the evaluated ELISA exhibits robust short-term repeatability. However, the storage time of extracts represents a significant and controllable source of negative bias. From an operational perspective, these findings emphasize that ELISA-based screening for ergot alkaloids should be performed on freshly prepared extracts whenever possible, or that extract stability must be validated if delayed analysis is unavoidable. This sensitivity to handling conditions is consistent with broader observations in the mycotoxin literature, where immunoassays can provide reliable precision under standardized workflows but remain vulnerable to preanalytical factors, particularly for structurally complex and chemically labile analytes such as ergot alkaloids.^6^

### 3.2 Intermediate Repeatability and Calibration Stability

Intermediate repeatability of the ELISA method was evaluated across 22 analytical runs conducted over 22 days, using independently prepared calibration curves and repeated analysis of an in-house wheat reference material (IHRM; approximately 75 μg/kg total ergot alkaloids, previously characterized by UHPLC-MS/MS). Across all runs, ELISA calibration curves generated with the kit-supplied standards exhibited excellent goodness-of-fit, with coefficients of determination (R^2^) ranging from 0.997 to 0.999. This high level of consistency demonstrates stable assay performance over time and confirms that the four-parameter logistic (4PL) model adequately describes the competitive binding behavior of the assay across the working concentration range. Such high calibration stability is characteristic of well-optimized competitive immunoassays, in which the sigmoidal response curve is primarily governed by antibody-analyte binding kinetics rather than instrumental variability. Similar calibration performance has been reported for commercial ELISA kits for other mycotoxins, including deoxynivalenol and zearalenone, with R^2^ values routinely exceeding 0.99 under controlled conditions.^28^

Analysis of the IHRM across the same 22 runs yielded an intermediate precision of approximately 6.0% RSD. Because the IHRM was freshly subsampled and extracted for each analytical run, this variability reflects the combined contributions of routine ELISA operational factors, including plate-to-plate differences, calibration curve preparation, incubation timing, and temperature control, as well as upstream sources such as ground-sample heterogeneity and extraction repeatability. For a screening method applied over multiple days, this level of variability is considered acceptable and aligns with performance ranges commonly reported for immunoassay-based mycotoxin screening methods, where interday RSD values of 5–15% are frequently observed.^28,31^

The stability observed here also supports the use of an in-house reference material as an effective quality control tool for ongoing ELISA monitoring. Similar approaches have been recommended in both regulatory laboratories and industry settings to ensure longitudinal consistency of screening assays, particularly when immunoassays are deployed alongside confirmatory UHPLC-MS/MS workflows.^6^

From a broader analytical perspective, these results reinforce a key principle highlighted in recent reviews of ergot alkaloid analysis. While immunoassays lack the compound-specific resolution of chromatographic methods.^1,32^ They can provide highly reproducible responses over time when assay conditions are tightly controlled. This reproducibility is essential for trend monitoring and preliminary screening, but must be interpreted with an understanding of the assay’s accuracy.

### 3.3 Accuracy Assessment Using Certified Reference Materials and Proficiency Test Samples

The accuracy of the commercial ELISA was evaluated using CRMs and PT samples representing different cereal matrices and ergot alkaloid profiles (Table 1). Publicly available product information for the ergot alkaloids ELISA describes the assay as detecting the sum of 12 major ergot alkaloids and reports strong agreement with assigned concentrations in the Bipea proficiency testing scheme.^25^ However, the extent to which such performance applies across different cereal matrices, naturally infested wheat samples, and variable alkaloid profiles remains important to evaluate independently. Therefore, the CRM and PT materials included here were used to compare ELISA performance with assigned values under the experimental conditions of the present study. Comparison of ELISA-derived total ergot alkaloid concentrations with certified or assigned values revealed variable agreement across materials, indicating that ELISA bias was not uniform across sample types or concentration ranges. Statistical comparison using one-sample tests was not applied because the objective was to assess ELISA accuracy relative to certified or assigned values, which themselves carry uncertainty, rather than to test whether replicate ELISA measurements differed from an error-free reference value. Therefore, accuracy was expressed as percent of the certified or assigned value and interpreted descriptively across materials. Across all reference materials evaluated, ELISA results ranged from 58% to 121% of the certified or assigned values, indicating substantial variability in method bias across matrices and concentration levels. For the lowest-level rye CRM (certified value 95.3 ± 4.7 μg/kg), the ELISA yielded a mean concentration of 55 ± 8 μg/kg, corresponding to an underestimation of approximately 42% relative to the certified value (Table 1). In contrast, for the midlevel rye CRM (certified value 508 ± 14 μg/kg), the ELISA result (491 ± 89 μg/kg) differed by only −3%, indicating close numerical agreement despite increased variability. At the highest level (certified value of 832.5 ± 46 μg/kg), the ELISA yielded a mean of 674 ± 91 μg/kg, indicating an average underestimation of approximately 19% (∼81% accuracy relative to the certified value), but with markedly elevated dispersion. At this level, the relative standard deviation exceeded 70%, compared with <15% for lower-level CRMs, indicating that variability increased disproportionately with concentration. The large standard deviation observed for this material suggests heterogeneity in immunoreactivity within the CRM, potentially reflecting an alkaloid distribution or epimeric composition that differs from the wheat cargo samples evaluated elsewhere in this study.

**Table 1:** Comparison of Total Ergot Alkaloid Concentrations Determined by ELISA for Certified Reference Materials (CRMs) and Proficiency Test (PT) Samples

Grain	Sample type (source)	Certified/accepted value (μg/kg)^a^	Mean ELISA result (μg/kg)	ELISA accuracy (% of certified value)
Rye	Certified reference material (Aokin)	95 ± 5	55 ± 8	58%
Rye	Certified reference material (Aokin)	508 ± 14	491 ± 89	97%
Rye	Certified reference material (Aokin)	833 ± 46	674 ± 91	81%
Wheat	Proficiency test sample (Bipea 04–0399)	241	291 ± 47	121%
Wheat	Proficiency test sample (Bipea 06–0399)	29,211	>4200	-

a Certified/accepted values were taken directly from the supplier certificates (for CRMs) and official PT reports (for Bipea samples). ELISA results are reported as mean ± standard deviation of N test portion replicate analyses.

Similar profile-dependent behavior has been reported for immunoassays targeting ergot alkaloids and other structurally diverse mycotoxins, including deoxynivalenol and zearalenone.^28,30^ In contrast to chromatographic methods, which quantify individual analytes using compound-specific response factors, ELISA results reflect an immunoreactivity-weighted total that depends on antibody affinity toward individual alkaloids and their relative abundance in the sample. As a consequence, samples with similar chromatographic totals can yield ELISA responses differing by several-fold, depending on the dominant alkaloids present. When the alkaloid profile of a CRM or PT sample differs markedly from the implicit profile used in the assay calibration, large positive or negative bias can result. This phenomenon has been discussed in method-comparison studies, where ELISA-derived totals may under- or overestimate chromatographic totals depending on the dominant alkaloids present.^30^ Overestimation of ergot alkaloids in rye and mixed cereal matrices has been reported in interlaboratory studies, underscoring the importance of interpreting ELISA results within the context of sample composition rather than solely on absolute numerical agreement.^33^

Taken together, the CRM and PT sample results indicate that the absolute accuracy of the commercial ELISA is matrix- and alkaloid-profile dependent rather than universally quantitative. For rye CRMs, ELISA accuracy ranged from approximately 58–97% of the certified values across the three materials, demonstrating acceptable agreement at moderate to high concentrations but systematic underestimation at the lowest level (∼95 μg/kg), consistent with reduced assay sensitivity near the lower end of the working range. In contrast, for the wheat PT samples, ELISA produced values higher than assigned (121% and >144%), suggesting a positive bias in matrices where the ergot alkaloid profile likely differs from the kit’s calibration basis. These patterns support the conclusion that the ELISA provides fit-for-purpose screening accuracy for identifying samples of potential concern, but that numerical agreement with UHPLC-MS/MS cannot be assumed for all matrices or concentration ranges.

From a regulatory and operational perspective, these results reinforce the necessity of confirmatory UHPLC-MS/MS analysis when ELISA results are used to support compliance decisions, particularly for samples near regulatory limits or for samples with alkaloid profiles that may differ from the ELISA calibration basis due to cereal species, season, geographic origin, fungal strain, storage, or processing history. At the same time, the variability observed across CRMs and PT materials highlights the value of ELISA as a rapid screening approach capable of identifying potentially high-risk samples even when absolute quantification is biased, provided that its composition-dependent response is clearly understood and integrated into a tiered analytical strategy. Similar conclusions have been drawn in recent evaluations of immunoassay performance for complex mycotoxin mixtures, which emphasize risk triage rather than absolute quantification.^6^

### 3.4 Comparison of ELISA and UHPLC-MS/MS Results

For method comparison, UHPLC-MS/MS was used as the chemical reference method, providing compound-specific quantification of individual ergot alkaloids and their epimers. A total ergot alkaloid concentration was calculated as the arithmetic sum of all quantified alkaloids included in the method. This summed value represents a chemically defined total based on compound-specific quantification of each analyte, rather than an immunoreactivity-weighted response governed by antibody affinity. As such, the UHPLC-MS/MS total serves as a chemically defined comparator against which the immunoreactivity-weighted ELISA response can be interpreted. Agreement between the commercial ELISA and the validated UHPLC-MS/MS reference method was evaluated using wheat and durum wheat cargo samples (*n* = 102) collected over multiple crop years (Figure 5). Linear regression analysis demonstrated a positive association between ELISA and UHPLC-MS/MS results across the full concentration range, indicating that the ELISA was responsive to increasing ergot alkaloid levels. However, the regression slope deviated substantially from unity, with ELISA results systematically lower than UHPLC-MS/MS values, indicating an approximate 2-fold underestimation. Bland-Altman analysis confirmed this proportional bias, showing a consistent negative mean difference between ELISA and UHPLC-MS/MS results across the concentration range (Figure 6). Importantly, the magnitude of disagreement did not increase randomly with concentration; rather, it reflected a systematic offset, indicating that the discrepancy was not driven by analytical imprecision or outliers but by fundamental differences in method response characteristics. This pattern indicates method-level bias rather than random analytical variability, which may arise from combined workflow effects including extraction, matrix response, and immunochemical cross-reactivity. The magnitude of the ELISA-UHPLC-MS/MS bias was substantially greater than the intermediate precision observed for the ELISA quality-control material, supporting the interpretation that the disagreement reflects systematic differences in method response rather than insufficient ELISA repeatability. Such behavior is well documented when immunoassays are compared with chromatographic methods for complex mycotoxin mixtures, including ergot alkaloids and other structurally diverse mycotoxins in cereals and feed.^28,30,33^ These studies consistently report matrix- and profile-dependent bias in ELISA relative to LC-MS/MS, arising from differential antibody cross-reactivity and the summed immunoreactivity response of the assay, because ELISA produces an immunoreactivity-weighted total that depends on antibody affinity toward individual alkaloids and their relative abundance in the sample, whereas UHPLC-MS/MS quantifies compound-specific concentrations of each alkaloid and its epimers using compound-specific transitions and response factors, and total ergot alkaloid concentration is calculated as the sum of these targeted analytes. In contrast, ELISA produces an immunoreactivity-weighted total, determined by antibody affinity toward individual alkaloids and their competitive binding behavior within the assay format. Consequently, agreement between ELISA and UHPLC-MS/MS depends not only on total concentration, but also on the relative distribution of individual alkaloids within each sample. Although differences in extraction solvent composition and pH may influence ergot alkaloid recovery, stability, and epimeric distribution, each method was applied according to its intended analytical workflow. The ELISA was performed using the manufacturer-recommended acidic methanol/water extraction, whereas UHPLC-MS/MS was performed using the validated acetonitrile/aqueous ammonium carbonate extraction procedure. Therefore, the comparison reflects the performance of the complete methods as used in practice, rather than an isolated comparison of extraction solvents. The sequential extraction experiment further showed that repeating the ELISA extraction under the same kit conditions did not substantially increase the ELISA-measured response, supporting the adequacy of the kit extraction within the ELISA workflow while recognizing that it does not directly compare acidic and ammonium carbonate-based extraction conditions. Previous ELISA-LC-MS/MS comparison studies for ergot alkaloids have reported both under- and overestimation depending on assay design and matrix, including differences in alkaloid profile, cereal type, and antibody cross-reactivity. Turner et al. observed substantial overestimation of ergot alkaloids by ELISA relative to HPLC in rye field samples, attributed to strong antibody cross-reactivity toward alkaloids prevalent in rye matrices. Similar profile-dependent bias has also been reported in interlaboratory and method-comparison studies,^33^ demonstrating that this behavior is not unique to a single data set.

**Figure 5:**
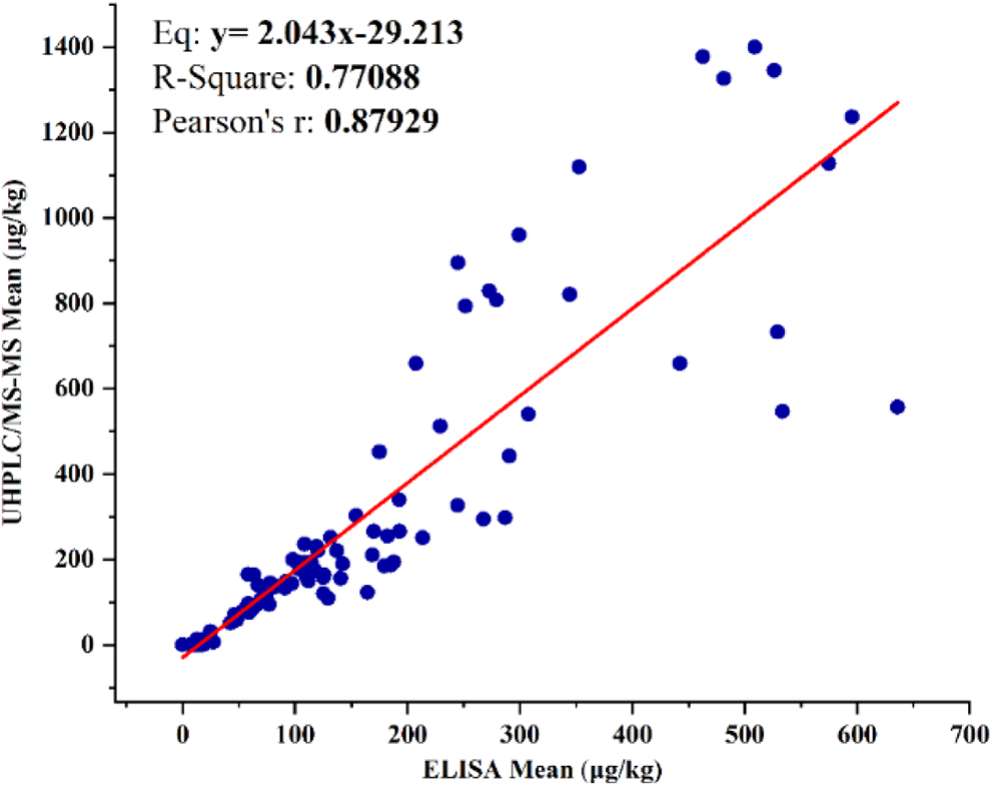
Linear regression analysis comparing total ergot alkaloid concentrations determined by ELISA and UHPLC-MS/MS for wheat and durum wheat cargo samples (*n* = 102). Each point represents the mean of duplicate measurements (extracted from the same test portion) ELISA determinations for an individual cargo sample, plotted against the corresponding UHPLC-MS/MS result. Statistical analysis was performed using linear regression.

**Figure 6:**
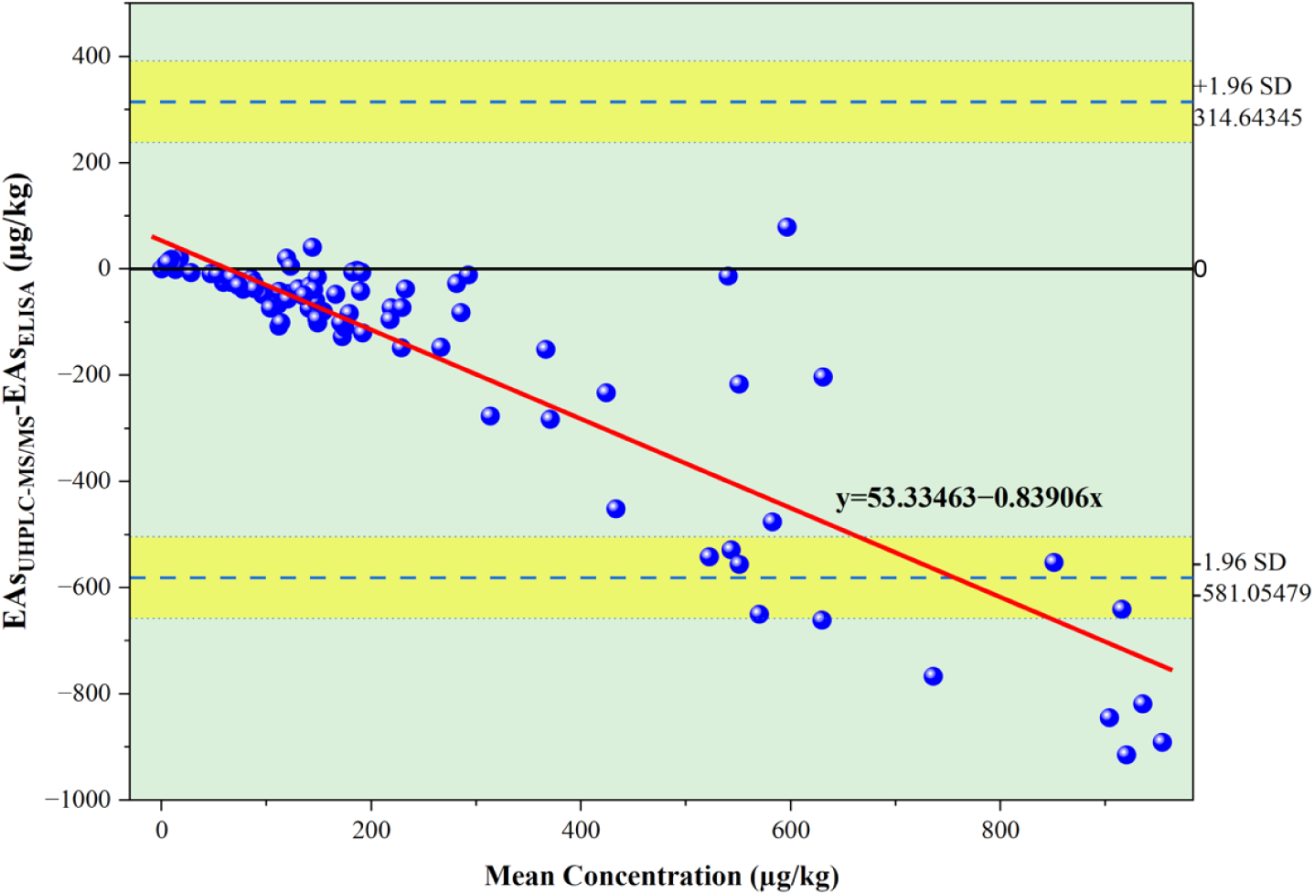
Bland-Altman plot comparing ELISA and UHPLC-MS/MS measurements of total ergot alkaloids for wheat and durum wheat cargo samples (*n* = 102).

Therefore, the systematic underestimation observed in the present study suggests that the wheat and durum samples analyzed were dominated by alkaloids and epimeric forms that elicited a lower antibody response under matrix conditions. Notably, because the regression includes a positive intercept (46.4 μg/kg), the relative bias is expected to be most pronounced at low-to-mid concentrations. In contrast, at higher concentrations, the proportional term (slope 0.38) dominates, consistent with the concentration-dependent ratio behavior shown in Figure 7. This interpretation is strongly supported by the cross-reactivity results presented in Table 2, which demonstrate wide variability in antibody response across individual ergot alkaloids and pronounced matrix-dependent effects. Reviews of ergot alkaloid analysis have emphasized that differences in antibody affinity for -ine versus -inine epimers can lead to substantial bias when immunoassays are used to estimate “total” ergot alkaloids.^1,18,32^ .

**Table 2:** Cross-Reactivity of Individual Ergot Alkaloids in Solvent and Wheat Matrix

Ergot alkaloid	Cross reactivity % vs ergotamine in solvent	Cross reactivity % vs ergotamine in wheat matrix extract
Ergometrine	56,854	1,825,100
Ergosine	129	7075
Ergotamine	100	100
Ergocornine	180	2177
Ergocryptine	17	43
Ergocristine	4	4043
Ergosinine	7	126
Ergocorninine	900	524
Ergocryptinine	150	14,150
Ergocristinine	90	472
Ergometrinine	4	33
Ergotaminine	21	177

**Figure 7:**
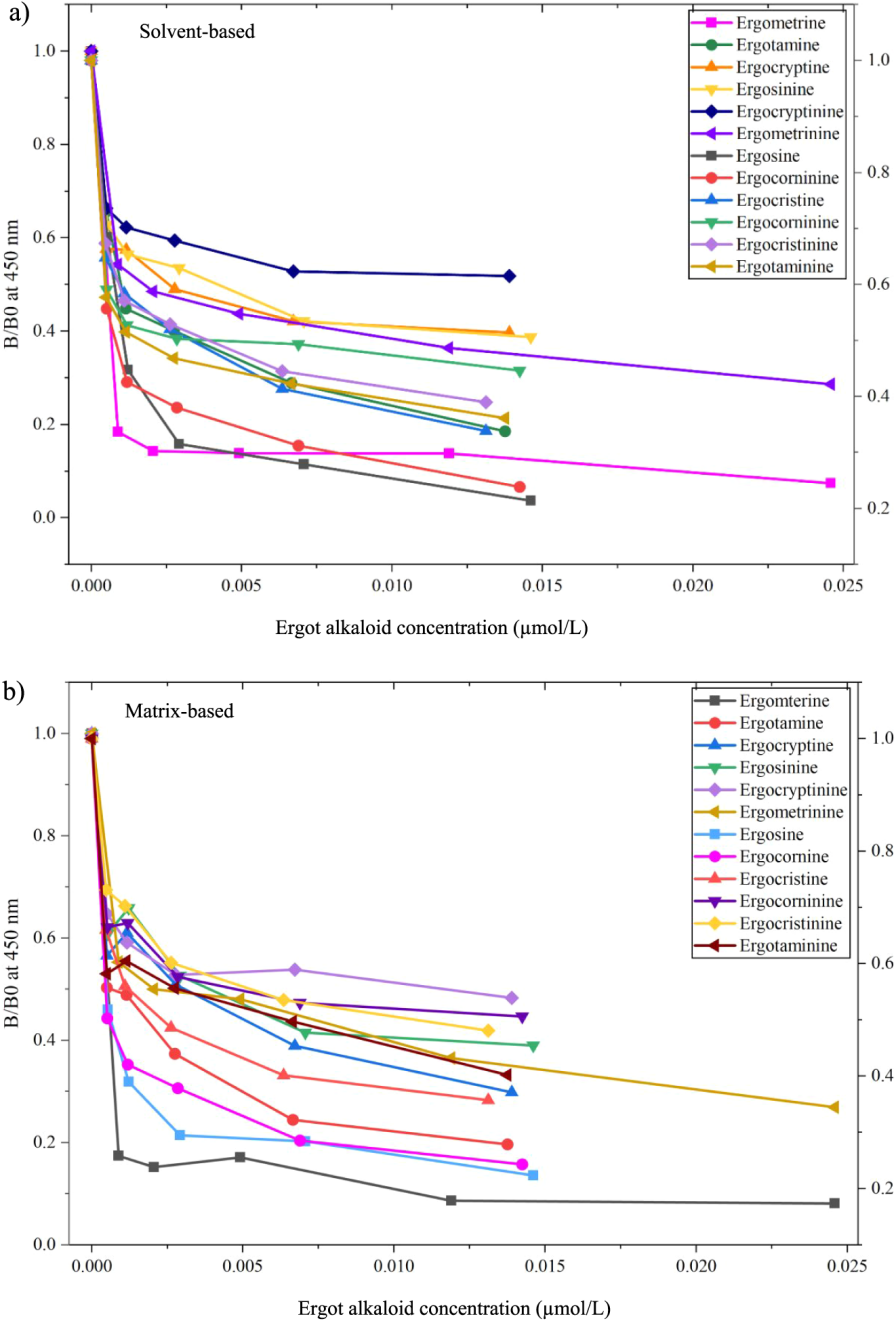
Relationship between the ELISA absorbance response, expressed as the ratio of absorbance relative to the blank (B/B_0_), where B refers to the optical density of the sample and B0 referring to the optical density of the blank, and the molar concentration of individual ergot alkaloids. (a) Solvent-based responses were obtained from individual ergot alkaloid standards prepared in the extraction solvent. (b) Matrix-based responses were obtained from the same ergot alkaloids spiked into a blank wheat extract and analyzed under identical conditions. Each data point represents the mean B/B_0_ of duplicate well measurements of the same standard level.

The strong correlation observed despite the bias indicates that the ELISA provides a consistent relative response to changes in ergot alkaloid concentration, making it suitable for ranking samples and identifying those requiring confirmatory analysis. Similar behavior has been reported for other mycotoxin ELISAs, where high correlation with LC-MS/MS is accompanied by systematic bias due to cross-reactivity and matrix effects.^28^ From a screening perspective, bias of this nature does not invalidate the use of ELISA, provided that decision thresholds are appropriately calibrated and that confirmatory UHPLC-MS/MS analysis is applied to samples of regulatory concern. The results presented in Figures 5 and 6, therefore, highlight a critical distinction between analytical agreement and analytical equivalence. While ELISA and UHPLC-MS/MS show strong agreement in trend and relative ranking, they are not analytically equivalent for the absolute quantification of total ergot alkaloids. Recognition of this distinction is essential for the appropriate interpretation of ELISA data in regulatory, trade, and risk assessment contexts.

### 3.5 Cross-Reactivity and Matrix Effects in the ELISA Response

The specificity of the commercial ELISA toward individual ergot alkaloids was systematically evaluated using an IC_50_-based cross-reactivity approach, following the principles established for immunoassay characterization by Tangni et al. (2010).^27^ Relative cross-reactivity was calculated using ergotamine as the reference compound and assessed both in solvent and in wheat matrix to explicitly evaluate matrix-dependent effects on antibody response (Table 2 and Figure 7a, b). In line with Tangni et al. (2010), this approach defines the “effective measurand” of the ELISA as an immunoreactivity-weighted signal rather than an equimolar total, which is essential for interpreting method-comparison outcomes.

In solvent, cross-reactivity varied significantly among the tested ergot alkaloids. Using ergotamine as 100% by definition, ergometrine exhibited extremely high cross-reactivity (56,854%). Moderate responses were observed for several compounds, including ergocorninine (900%), ergocornine (180%), and ergocryptinine (150%), while ergosine was near unity (129%). Several alkaloids, including ergocryptine (17%), ergocristine (4%), ergometrinine (4%), ergotaminine (21%), and ergosinine (7%), exhibited minimal response under solvent conditions. The range of cross-reactivity observed demonstrated that antibody binding was highly compound-dependent, even within closely related ergot alkaloids and epimeric pairs. Comparison of corresponding -ine and -inine epimer pairs showed that epimerization strongly affected antibody recognition. In solvent, several epimer pairs differed markedly in cross-reactivity, including ergosine/ergosinine (129% vs 7%), ergotamine/ergotaminine (100% vs 21%), ergocryptine/ergocryptinine (17% vs 150%), and ergocristine/ergocristinine (4% vs 90%). These differences indicate that the antibody response is not governed only by the shared ergoline backbone or molecular mass, but also by stereochemical configuration and side-chain structures.

When the same alkaloids were tested in a wheat matrix, cross-reactivity increased markedly for many compounds, with amplification magnitude strongly analyte-dependent (Table 2 and Figure 7b). The most extreme case was ergometrine, which increased from 56,854% in solvent to 1,825,100% in wheat matrix relative to ergotamine. This shift moved ergometrine from a midranked immunoreactive alkaloid in solvent to the dominant contributor to the ELISA signal in wheat matrix. Similarly strong matrix amplification was observed for ergocryptinine, ergocristine, and ergosine, with cross-reactivities increasing 55- to 1000-fold, collectively elevating these analytes from minor to major contributors to the overall ELISA response in matrix. Some analytes remained weakly immunoreactive even under matrix conditions, including ergometrinine (33%) and ergocryptine (43%). The epimer-dependent response became even more pronounced in wheat matrix, where some pairs showed large shifts in relative response, such as ergocryptine/ergocryptinine (43% vs 14,150%) and ergometrine/ergometrinine (1,825,100% vs 33%). Importantly, the relative ranking of individual alkaloids changed between solvent and matrix: ergocorninine dropped from one of the most immunoreactive compounds in solvent (∼900%) to a midranked species in matrix (524%), whereas ergocristine rose from among the least reactive alkaloids in solvent (4%) to a top-ranked contributor in matrix (4,043%). This demonstrates that matrix effects can both amplify and suppress analyte responses and can fundamentally reweight which alkaloids dominate the ELISA signal. These opposing trends demonstrate a matrix-driven reordering of which alkaloids dominate the ELISA signal, rather than a uniform scaling effect. Therefore, matrix-dependent cross-reactivity spanned more than 4 orders of magnitude, ranging from 33% (ergometrinine) to 1,825,100% (ergometrine) in wheat matrix, compared with 4–56,854% under solvent conditions, demonstrating that ELISA response is dominated by compound- and matrix-specific antibody affinity rather than equimolar contribution of individual ergot alkaloids. Taken together, these shifts in relative ranking confirm that matrix effects can fundamentally reshape which ergot alkaloids drive the ELISA response.

These findings closely parallel those reported by Tangni et al. (2010) for deoxynivalenol immunoassays, in which antibody cross-reactivity toward structurally related fusariotoxins varied widely and was strongly influenced by the assay environment. In that study, cross-reactivity toward acetylated and conjugated DON derivatives resulted in substantial overestimation relative to LC-MS/MS, particularly when these derivatives co-occurred in cereal matrices. Tangni et al. emphasized that cross-reactivity is not an analytical artifact but an inherent property of antibody-based detection that must be explicitly characterized and periodically reevaluated. Consistent with that framework, the present data show that “total ergot alkaloids” by ELISA is not a fixed chemical measurand: it depends on antibody affinity and matrix context. It is therefore sensitive to changes in the distribution of alkaloids (analog/epimer composition) between samples.

The B/B_0_ versus molar concentration plots further illustrate these effects. In solvent (Figure 7a), absorbance ratios for different ergot alkaloids followed broadly similar declining trends with increasing concentration, indicating relatively consistent antibody-analyte interactions in the absence of coextracted matrix components. In contrast, responses in wheat matrix (Figure 7b) exhibited substantially greater divergence among analytes at comparable concentrations, with some compounds showing pronounced signal suppression and others showing marked amplification. Because each point represents a mean B/B_0_ response, the wider spread among analytes in the matrix indicates that compounds at similar concentrations can yield very different immunochemical responses when the matrix is introduced, supporting the conclusion that chemical structure alone is insufficient to predict ELISA behavior. Similar matrix-induced alterations in immunoassay response have been widely documented and are attributed to changes in analyte availability, antibody-hapten interaction kinetics, and nonspecific interactions with coextracted matrix components.^27,34–36^

From an analytical perspective, the solvent-versus-matrix contrast observed here provides a direct mechanistic explanation for the bias observed between ELISA and UHPLC-MS/MS measurements. Although matrix effects can also occur in UHPLC-MS/MS through ion suppression or enhancement, these effects can be evaluated and mitigated using internal standards, matrix-matched calibration, dilution, cleanup, or standard addition. In contrast, ELISA matrix effects arise from changes in antibody-analyte interactions, analyte availability, and nonspecific binding, and are therefore more difficult to correct universally; mitigation typically relies on matrix-matched calibration, optimized extraction and dilution, validation in the relevant matrix, and confirmatory chromatographic analysis. UHPLC-MS/MS quantifies individual ergot alkaloids and reports their arithmetic sum, whereas the ELISA integrates an immunochemical signal weighted by antibody affinity and matrix effects. Consequently, samples dominated by weakly immunoreactive alkaloids will be underestimated, whereas samples enriched in highly cross-reactive compounds may be substantially overestimated. For example, if ergometrine contributes materially to a sample’s true total, its matrix CR of 1,825,100% indicates it can disproportionately drive ELISA signal; conversely, if a sample is enriched in weakly responding analytes such as ergometrinine (33%) or ergocryptine (43%), the ELISA would be expected to yield a much lower apparent “total” relative to the chromatographic sum. Similar bidirectional bias has been reported for immunoassays targeting ergot alkaloids.^30^ Importantly, the magnitude and variability of cross-reactivity observed in this study extend that finding by showing how strongly the wheat matrix can reshape the ELISA signal. Across the panel, matrix CR values ranged from 33% to 1,825,100%, whereas solvent CR values ranged from 4% to 56,854%, confirming that the ELISA response function is highly nonuniform across analytes and is further modulated by the matrix.

Together, these data add a new mechanistic layer to the comparison: they show that disagreement between ELISA and UHPLC-MS/MS is not only due to differences in summation versus compound-specific quantification (discussed earlier in this section), but also to matrix-dependent reweighting of individual alkaloids through antibody cross-reactivity. This means that ELISA-derived “total ergot alkaloids” should not be interpreted as chemically equivalent to chromatographic totals. Instead, the assay provides a rapid screening estimate that reflects the combined effects of antibody cross-reactivity, relative alkaloid composition, and matrix influences, rather than a true stoichiometric sum of individual EA compounds. In addition, the cross-reactivity data highlight the necessity of matrix-specific validation and careful interpretation of ELISA results for ergot alkaloids. The observed matrix amplification factors (e.g., ∼32× for ergometrine, ∼94× for ergocryptinine, ∼55× for ergosine, and ∼1,000× for ergocristine) demonstrate that “one-size-fits-all” calibration assumptions are not valid when alkaloid profiles vary between matrices. The compound- and matrix-dependent variability observed reinforces the current consensus that positive ELISA findings should be confirmed by compound-specific UHPLC-MS/MS analysis, particularly for samples near regulatory decision thresholds or exhibiting atypical alkaloid profiles.

Taken together, the results demonstrate that the evaluated ELISA provides robust repeatability and calibration stability under controlled conditions, supporting its operational suitability for high-throughput screening of wheat for ergot alkaloids. The lack of significant effects from lot and extraction thoroughness, coupled with stable 4PL calibration performance across multiple analytical days, indicates that procedural variability is not the primary source of disagreement with UHPLC-MS/MS. Instead, the cross-reactivity data (Table 2) provide a mechanistic basis for why strong correlation can coexist with systematic bias: the ELISA response is intrinsically reweighted toward (or away from) specific alkaloids/epimers depending on matrix context.

Operationally, the demonstrated sensitivity of ELISA response to extract storage highlights the need for strict adherence to handling protocols. Although the commercial kit is marketed for several cereal matrices and milling products, the present solvent-versus-wheat matrix cross-reactivity results show that ELISA response can be strongly matrix dependent. Therefore, matrix-relevant validation remains important when applying the assay across cereal types or processed products. Therefore, the integrated evidence supports deploying the ELISA within structured monitoring programs, while clearly outlining the circumstances under which UHPLC-MS/MS confirmation remains essential for regulatory compliance and risk management.

This study provides a comprehensive evaluation of a commercially available ELISA for total ergot alkaloids, using wheat samples as the primary application matrix, and integrates performance metrics with mechanistic insights into method behavior. While the assay demonstrated strong repeatability, calibration stability, and robustness to procedural factors such as test portion mass and extraction conditions, systematic differences relative to UHPLC-MS/MS were consistently observed. By linking these differences to compound-specific antibody cross-reactivity and matrix-dependent signal modulation, this work clarifies that ELISA-derived results reflect an immunochemically weighted response rather than a stoichiometric total. This distinction is critical for the appropriate interpretation of ELISA data and supports its use within tiered analytical strategies as a rapid screening tool, complemented by UHPLC-MS/MS for confirmatory quantification and regulatory decision-making.

## Abbreviations

EA(s): ergot alkaloid(s)
ELISA: enzyme-linked immunosorbent assay
UHPLC-MS/MS: ultrahigh-performance liquid chromatography-tandem mass spectrometry
LC-MS/MS: liquid chromatography-tandem mass spectrometry
CRM(s): certified reference material(s)
PT: proficiency test
IHRM: in-house reference material
RSD: relative standard deviation
SD: standard deviation
R^2^: coefficient of determination
4PL: four-parameter logistic
IC_50_: half-maximal inhibitory concentration
B/B_0_: ratio of sample absorbance to blank absorbance
HPLC: high-performance liquid chromatography
UHPLC: ultrahigh-performance liquid chromatography
MRM: multiple reaction monitoring
ESI: electrospray ionization
CGC: Canadian Grain Commission
DON: deoxynivalenol.
